# Moon (or Eid) Crescent Sign of the Femoral Head

**DOI:** 10.7759/cureus.6867

**Published:** 2020-02-04

**Authors:** Sattar Alshryda, Haidar Alfuqaha, Elham A Elgabaly, Ehab Aldlyami

**Affiliations:** 1 Pediatric Orthopaedics and Trauma, Al Jalila Children's Speciality Hospital, Dubai, ARE; 2 Pediatric Orthopaedics, Alkhalidi Hospital, Amman, JOR; 3 Pediatric Radiology, Al Jalila Children Specialty Hospital, Dubai, ARE; 4 Trauma and Orthopaedics, Medcare Orthopaedics and Spine Hospital, Dubai, ARE

**Keywords:** scfe, sufe, avn, slipped, hip, avascular, necrosis

## Abstract

Avascular necrosis (AVN) of the femoral head following slipped capital femoral epiphysis (SCFE) is a serious complication that often leads to a permanent disability. Radiological findings of AVN may take up to two years to become apparent. This means painful waiting for children, parents, and treating teams. We would like to describe a new radiological sign that we noted in four patients. The sign has been named as the crescent moon sign or eid crescent sign. It may become visible as early as six weeks following surgery, and it carries a good prognosis that the femoral head is viable and will not develop AVN. Two out of the four patients were treated in our hospital by Ganz surgical dislocation. The other two patients had been featured in other publications, but the significance of the moon crescent signs, which were present, was not recognized or appreciated. All four patients did not develop AVN. A relatively similar radiological sign has been described in talus bone fractures (Hawkins' sign). Like SCFE, talus bone fractures have a high AVN rate. Both, the crescent moon sign and Hawkins' sign carry a good prognosis and indicate that the bone blood supply is restored.

## Introduction

Slipped capital femoral epiphysis (SCFE) is one of the important pediatric orthopedic conditions. It affects one in 100 000 children a year. Loder classified SCFE into two types: stable and unstable SCFE. Patients with stable SCFE are able to walk and the risk of avascular necrosis (AVN) is low; however, patients with unstable SCFE are not able to walk even with crutches. The risk of AVN is high [1]. SCFE can be also classified as mild, moderate and severe based on the degree of femoral head displacement in relation to the neck. The current trend is to treat mild and moderate SCFE by stabilization without reduction; however, there is increasing evidence to support open reduction and stabilization of severe SCFE [2, 3]. AVN is one of the most serious complications of SCFE; particularly the unstable type. It occurs because of loss of blood supply to the femoral head. AVN may not be radiologically visible for up to two years - a long period of waiting [3]. We describe a new radiological sign that carries a good prognosis in four patients with slipped capital femoral epiphysis. Two patients were treated by our team, whereas the other two were reported in previous publications [4, 5]. Their X-rays showed the crescent moon sign that we describe in this report, but publishing authors did not appreciate its importance. 

## Case presentation

Our patients presented with severe, stable slipped capital epiphysis. They underwent surgical correction using the Ganz surgical dislocation technique. During surgery, the patients were placed in a lateral with the affected side upward. Bony prominences were padded to prevent pressure sores. Fluoroscopy screening was performed prior to preparation to ensure optimal visualization of the hip. After preparation and draping, a 6 cm incision was made over the greater trochanter extending from the iliac crest to the lower part of the greater trochanter. The perforators of the inferior gluteal artery were identified to dissect the plane between gluteus maximus and tensor fascia lata muscles. Then the posterior border of the gluteus medius and the anterior border of piriformis muscles were identified and separated to expose the joint capsule. A 15 mm thick trochanteric osteotomy was then made with a power saw and osteotome. The osteotomised trochanter was mobilized anteriorly with the attached Glutei medius and minimus to expose the capsule fully.

A Z-shaped capsulotomy was then performed while protecting the femoral head and the labrum. This was carried distally along the intertrochanteric line to the lesser trochanter and proximally along the acetabular margin to the piriformis muscle. The femoral epiphysis was then stabilized with two K-wires to avoid further slippage during the dislocation. The vascularity of the femoral head was assessed by making a 2-mm drill hole to the femoral head. 

The ligament teres was divided using the long curved scissor to allow for safe hip dislocation and inspecting the femoral head and acetabulum for any damage. Then the femoral head was carefully dissected subperiosteally with a long retinaculum flap (which has the vascular supply for the femoral head). The remaining physis was curetted from the epiphysis, which was then reduced into the metaphysis. The femoral head was then stabilized using two cannulated screws. 

The hip joint was then reduced and the retinaculum and joint capsule were loosely closed to avoid constrictive effect on the retinaculum vessels. The trochanteric osteotomy was reduced back to its bed and stabilized with two screws. Skin layers were closed with absorbable sutures.

**Figure 1:**
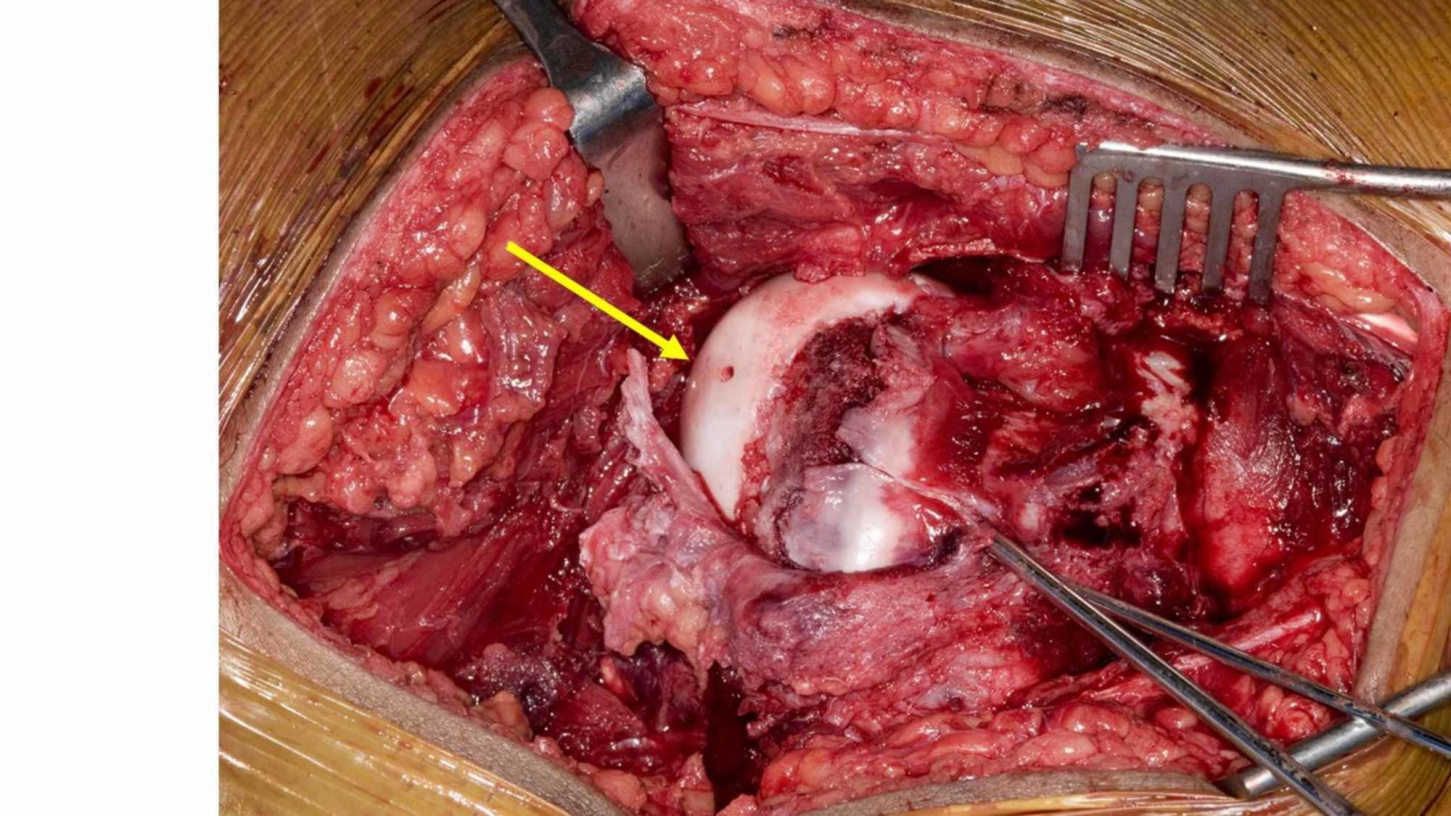
Intraoperative clinical photograph of the femoral head The intraoperative clinical photograph shows the femoral head during surgical dislocation in a child with a slipped capital epiphysis. The femoral head was drilled (yellow arrow) and there is no bleeding from the drill hole. This is a bad clinical sign which was associated with future avascular necrosis.

**Figure 2:**
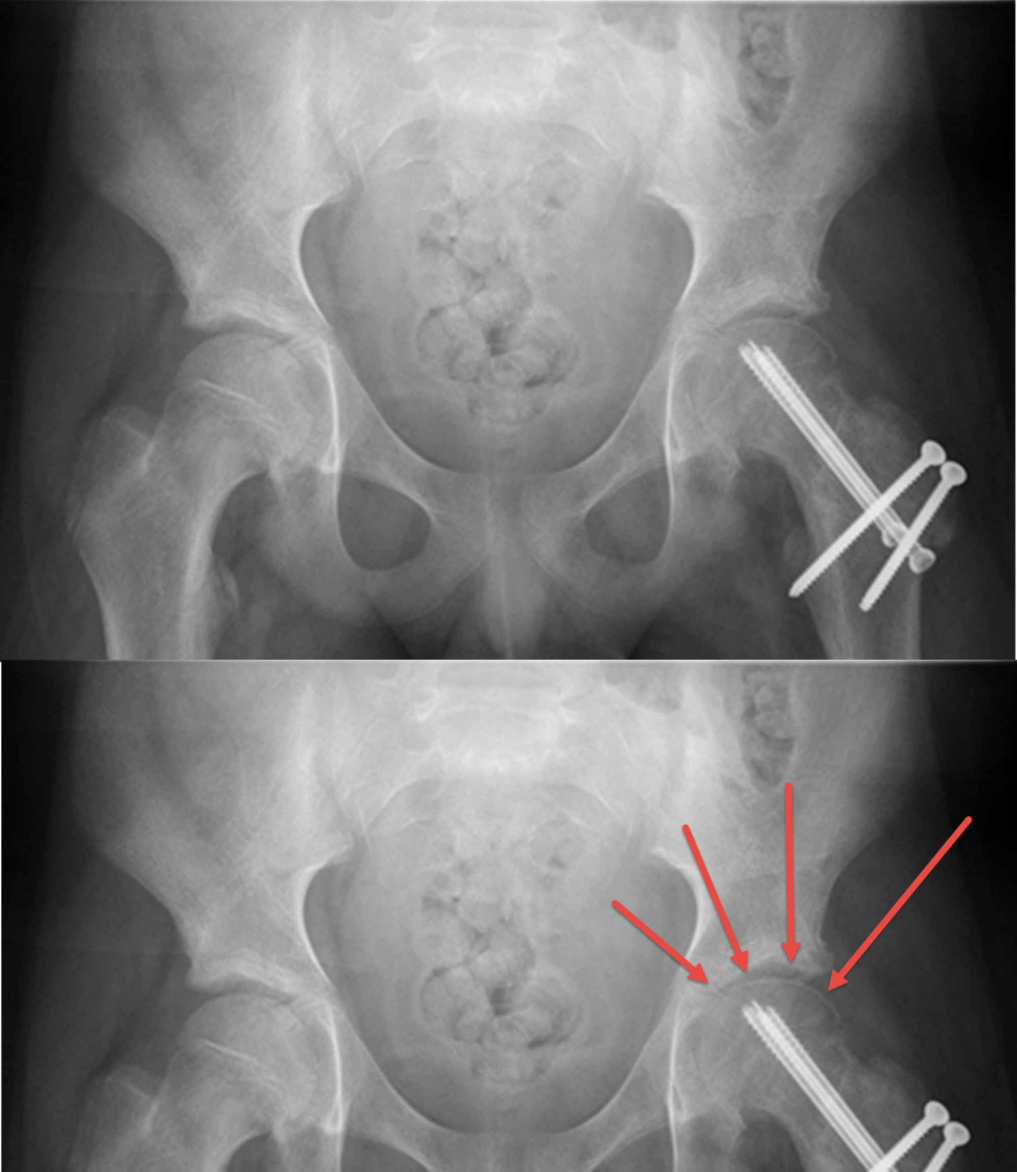
A pelvis x-ray moon or eid crescent sign Top image: postoperative plain pelvis x-ray (anteroposterior view) showing the anatomical reduction and the stabilizing screws. There is subchondral crescent-like osteopenia (moon crescent sign). Bottom image: close up image of the femoral head with the red arrows highlighting the subchondral crescent-like osteopenia (moon crescent sign).

To sum up, two patients presented to our department with severe unstable SCFE. Both were treated by Ganz surgical dislocation which involved reducing the femoral head to its normal position using the flip trochanteric osteotomy [6]. Intraoperatively, the femoral head was drilled using a 2 mm drill bit and there was no bleeding of the femoral head (Figure 1). This is considered a poor prognostic sign and the likelihood of future AVN in such patients is very high. However, the “crescent moon” radiological sign was noted in both patients within six weeks after surgery (Figure 2). Both hips went to heal fully with no AVN.

**Figure 3:**
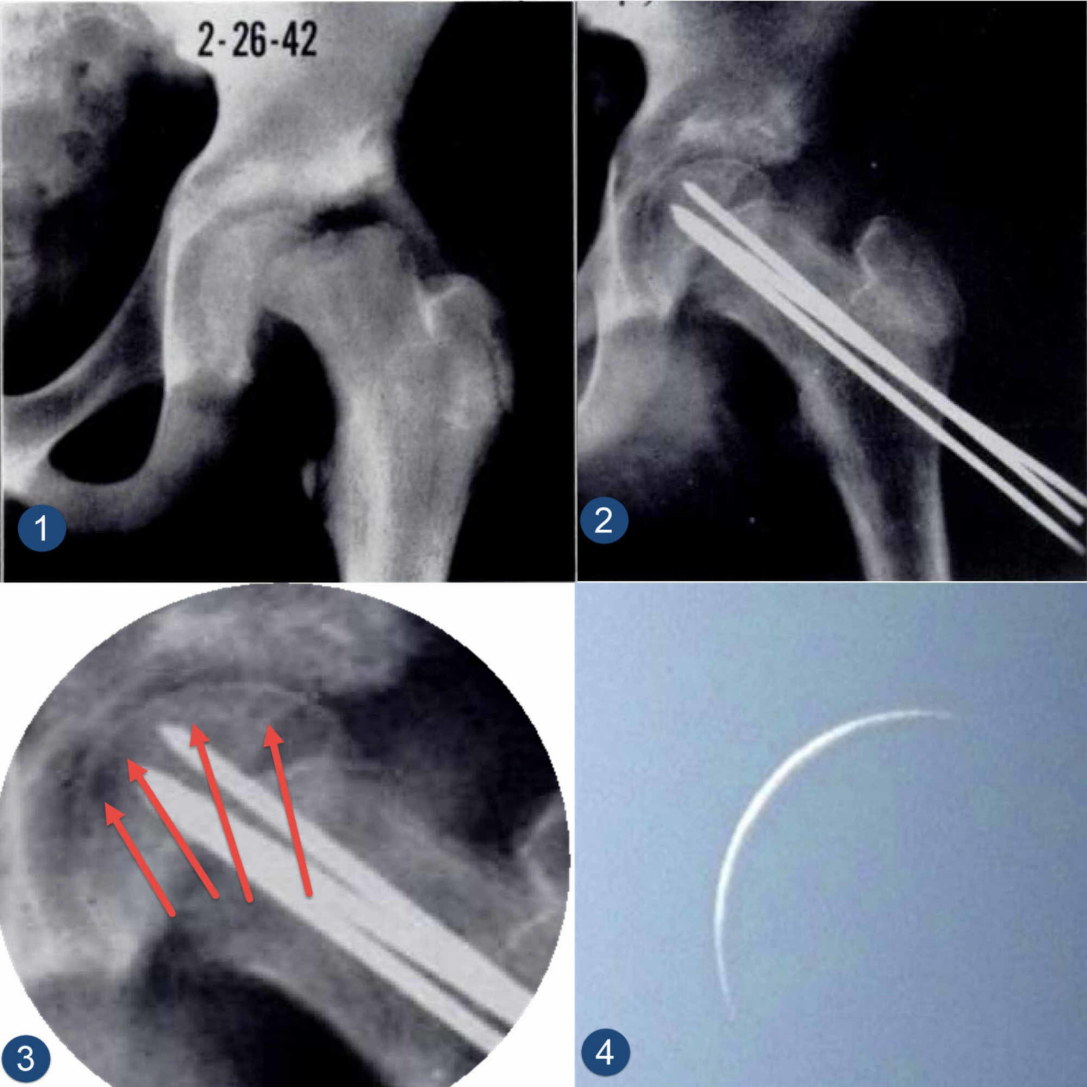
Crescent moon sign in a child following slipped capital femoral epiphysis stabilization Image 1 (left top image): a preoperative plain radiograph of the left hip of a child who presented with a severe slipped capital femoral epiphysis who was treated with a gentle reduction and stabilization. Image 2 (right top image): a postoperative plain radiograph of the child's hip showing a good reduction. Three smooth K-wires were used to stabilize the slip. There is a visible subchondral crescent-like osteopenia (crescent moon sign). Image 3 (left bottom image): a close-up image of the left femoral head of the hip highlighting the subchondral crescent-like osteopenia (crescent moon sign) with four red arrows. Image 4 (right bottom image): a photograph of the crescent moon, taken at the beginning of the lunar month to illustrate the similar shape of the crescent moon to the radiological appearance of the femoral head when revascularisation happens.

**Figure 4:**
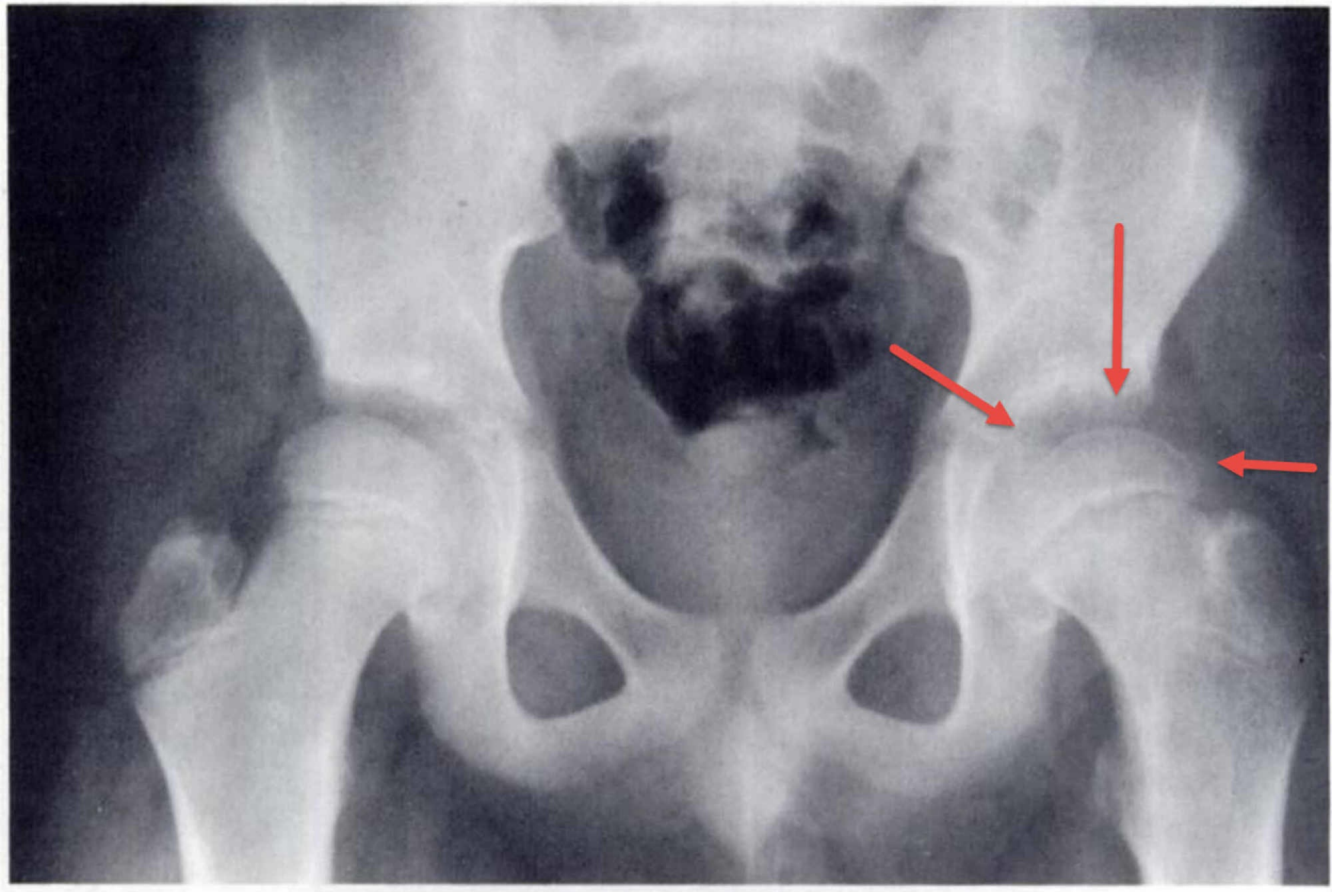
Crescent moon sign in a child with slipped capital epiphysis who was treated with a hip spica A plain radiograph of an eight-year-old child with a slipped capital femoral epiphysis who was treated with a hip spica. The red arrows point toward the subchondral crescent-like osteopenia (moon crescent sign). The patient did not develop avascular necrosis of the femoral head.

Fahey et al. and Betz et al. independently published postoperative x-rays of two children with SCFE showing the crescent moon sign (Figures 3 and 4) [4, 5]. Both children did not develop AVN. Fahey reported on a 14-year-old boy who was admitted after falling on the ice. The slipped was reduced closed, unlike our patient, and stabilized with three partially threaded pins. A closed reduction of SCFE has been shown to increase the risk of AVN further. The two-month postoperative x-ray showed the crescent moon sign but it was not known then. The child never developed AVN as shown by his pelvis x-ray after 20 years. Betz reported on an eight-year-old boy who presented with acute on chronic grade I slip. He was successfully treated with a cast for 12 weeks and did not develop AVN.

## Discussion

Avascular necrosis of the femoral head is one of the most serious complications in children because there is currently no effective treatment. Several authors proposed some predictors for the development of the femoral head AVN, but none is absolute [2, 3, 7]. Therefore, the postoperative period usually turns into a time of painful suspense.

The X-rays of the four cases that we described showed subchondral crescent-like osteopenia (crescent moon sign) on X-ray, which is a sign of revascularisation. A similar sign (Hawkins' sign) is well described in the talus bone neck fractures in which the bone AVN is very common [8, 9]. To the best of our knowledge, this sign has not been described in any other part of the human body. Its presence indicates that revascularisation has occurred and that the AVN is unlikely.

This sign has not been reported following SCFE before. However, the two aforementioned papers included X-ray images with the sign clearly visible. Both papers indicated that the relevant patients did not develop AVN. Fahey and colleagues, as part of a case series, published on a child with SCFE who underwent gentle reduction and stabilization. Two months later, the X-ray showed clear subchondral bone osteoporosis (crescent moon sign) (Figure 3) [5]. Betz and colleague reported on another eight-year-old child with SCFE who was treated with a hip spica. His x-ray showed obvious subchondral crescent-like osteopenia (crescent moon sign) (Figure 4) [6]. Both of these patients did not develop AVN. The authors did not recognize this sign or its similarity to the Hawkins' sign that was described in talus bone fractures.

The name of "crescent moon sign" has been adopted from its obvious similarity to the crescent moon (Figure 3 image 4); however, we prefer the name "eid crescent sign" which is adopted form the Muslim lunar calendar for three reasons: the close similarities between the shape of the crescent moon at the beginning of the month and the shape of the subchondral crescent-like osteopenia (crescent moon sign) (Figure 3 image 4). People search attentively to see the crescent moon, which denotes the beginning of Eid (a major Muslim festival) and celebration. The sight of the crescent moon brings lots of happiness. This is very similar to our attentive search for this subchondral crescent-like osteopenia (the Eid crescent sign), which brings happiness to all when it is seen.

## Conclusions

The slipped capital femoral epiphysis is not a common condition, and if severe, it requires reduction and surgical stabilization. Avascular necrosis is one of the devastating complications that can occur following treatment. This may take months or even years to become visible on plain radiographs. We have described a novel radiological sign that is visible as soon as six weeks following surgery, and it carries a good prognosis. We named it - the Eid crescent sign. We encourage colleagues to actively search for this sign following SCFE treatment and report it to validate its significance.
